# Does Multi-Strain Probiotic Supplementation Impact the Effort Capacity of Competitive Road Cyclists?

**DOI:** 10.3390/ijerph191912205

**Published:** 2022-09-26

**Authors:** Paulina Mazur-Kurach, Barbara Frączek, Andrzej T. Klimek

**Affiliations:** 1 Department of Physical Education and Sport, Institute of Biomedical Sciences, Faculty of Sports Medicine and Human Nutrition, University of Physical Education, Jana Pawła II 78, 31-571 Kraków, Poland; 2 Department of Physical Education and Sport, Institute of Biomedical Sciences, Faculty of Physiology and Biochemistry, University of Physical Education, Jana Pawła II 78, 31-571 Kraków, Poland

**Keywords:** athletes, performance, sport, probiotics, health

## Abstract

(1) Background: The aim of this study was to assess the impact of multi-strain probiotic supplementation on the physical capacity and selected health indicators related to the exercise capacity of competitive road cyclists such as body composition, markers of intestinal permeability, pro- and anti-inflammatory markers, and anti-/pro-oxidant potential. (2) Methods: The group comprised 26 competitive road cyclists aged between 18 and 26. The study was a 4-month double-blind, random-assignment, parallel-group, and placebo-controlled trial. The measurements of physical capacity in the exercise tests of the anaerobic Wingate test (the level of total work volume, maximal anaerobic power, average power per revolution, mean time to achieve maximal anaerobic power, and time to maintain maximal anaerobic power) and the aerobic test using a cycle ergometer (maximum oxygen uptake, exercise duration, maximum load power, and maximal heart rate) were repeated after one, three, and four months. (3) Results: The probiotic supplementation resulted in increased levels of the relative magnitude of maximal oxygen uptake (65.28 vs. 69.18), the duration of training until failure (14.35 vs. 15.65), the load on the ergometer (5.11 vs. 5.36), and the degree of decrease in heart rate (193.3 vs. 188.6) together with a feeling of less discomfort during the exercise test (Borg scale) (19.38 vs. 18.43), confirming the beneficial effect of probiotics on the cyclists’ aerobic capacity during exercise. The probiotic supplementation produces no effects on the anaerobic capacity and body composition of the athletes, except for an observed increase in muscle mass. The concentration of zonulin in the stool mass decreased as a result of the probiotic therapy (81.2 vs. 25.21), and α1-atitrypsin was maintained at a similar level during the experiment (0.95 vs. 1.05), indicating a sealing of the intestinal barrier and beneficial changes in the cyclists’ intestinal function. The supplementation resulted in a reduction in the concentrations of: tumor necrosis factor TNF-α after the aerobic (13.88 vs. 9.75) and anaerobic tests (8.54 vs. 6.8), IL-6 before (1.2 vs. 0.86) and after the anaerobic test (1.47 vs. 0.97), IL-10 before the anaerobic test (0.70 vs. 0.44), and the total oxidative status (TOS) of the blood plasma before (663.7 vs. 484.6) and after the anaerobic test (643.1 vs. 435.9). (4) Conclusions: The probiotic supplementation resulted in increased levels of the cyclists’ aerobic capacity and their maintenance of anaerobic capacity and positively affected selected health indicators related to the exercise capacity of competitive road cyclists.

## 1. Introduction

Strenuous and prolonged exercise induces stress on the gastrointestinal tract (GI) that increases the likelihood of multiple symptoms associated with disturbed gut microbiota and decreased performance [1], including abdominal cramping, acid reflux (heartburn), nausea, vomiting, diarrhea, and the permeability of the gut, which may precipitate systemic endotoxemia [2,3,4,5]. Intense exercise in athletes, as individuals exposed to other multiple environmental factors (i.e., psychological stress, inadequate diet, energy deficiencies, time zone changes, disrupted sleep habits, exposure to an abnormal external environment, and the intake of medications), especially in the absence of adequate recovery time, can lead to the disruption of the microbiota (dysbiosis) and the increased permeability of the intestinal barrier [6,7,8,9,10,11,12]. Dysbiosis fosters a decrease in the synthesis of proteins that are structural elements of gap junctions between intestinal epithelial cells (zonulin-1 and occludin). This causes functional damage to the integrity of the gastrointestinal mucosa and the migration of harmful substances into the blood [13,14,15,16,17]. Probiotics are living nonpathogenic microorganisms administered to improve microbial balance, particularly in the gastrointestinal tract. They may regulate the mucosal immune response [18], improve the activity of macrophages [18], and modulate the expression of the genes associated with macrophage activity. Probiotics may also interact with Toll-like receptors (TLRs) and downregulate the expression of nuclear factor (NF)-κB and proinflammatory cytokines [19,20]. Additionally, the levels of anti-inflammatory cytokines and immunoglobulins, immune cell proliferation, and the production of proinflammatory cytokines by T cells may be modulated following probiotic supplementation [21,22]. Probiotic supplementation may modify the gut microbiota composition, promoting increased microbial diversity and supporting the growth of health-promoting species [23,24,25]. Probiotics may also help restore a disturbed gut microbiota [26] and support microbiota under stress [27,28]. There is evidence from studies using probiotics that show potential positive effects in athletes [14,29,30,31,32,33,34,35,36,37,38,39]. The possible mechanisms of action include: (1) enhancing the natural barrier function of the normal intestinal mucosa, (2) the modulation of the immune system, (3) the antagonism of pathogens, and (4) the production of enzymatic activities and beneficial metabolites for the host. According to certain studies, probiotics may benefit athletes indirectly by maintaining gastrointestinal function and health, which prevents the immunosuppressive effects and respiratory tract infections caused by intense exercise. This in turn reduces athletes’ susceptibility to illness and consequently improves athletic performance [40]. The microbiome may also have an indirect functional influence on various indices of exercise performance and recovery [30,31,32,33,34,35,36,37,38,39,40,41]. Therefore, probiotics as functional modulators of the microbiome can potentially promote health, exercise adaptation, and performance in athletes. Given the current knowledge, this study evaluated the effectiveness of probiotic supplementation on the physical capacity and selected health indicators related to the exercise capacity of competitive road cyclists such as body composition, markers of intestinal permeability, pro- and anti-inflammatory markers, and anti-/pro-oxidant potential. This study could assist further research into the effects of probiotic supplementation on the development of dietary guidelines for athletes.

## 2. Materials and Methods

This research involved 26 road cyclists, representing clubs from the Małopolska region, who regularly and successfully participate in domestic and foreign competitions. The main criteria for inclusion in the studied group were experience in competitive cycling for at least 5 years, good health (assessed during medical qualification), male sex, age between 18–26 years, and a high sporting level (participation in national and international cycling competitions). The average training period of the athletes was over 9 years. Participants on immuno-modulatory medications were excluded. Inclusion in the study was dependent on the subjects not taking antibiotics or supplements/foods containing probiotics for at least one month prior to and during the study period. The athletes were trained by two coaches. The coaches worked closely together and planned the training regimen for this study. Training loads were the same for all cyclists. The cyclists undertook 1.48 ± 0.8 training sessions every day lasting approx. 104.28 ± 5.34 min, which accounted for about 6 training units during the week, with a total length of approx. 11 h.

The experiment was a randomized, double-blind study. The athletes were assigned to a group taking probiotic supplements (P) or a control group taking a placebo (C). The athletes’ measured physical parameters are as follows (given as means): age of the athletes was 23.25 years (P) vs. 21.28 (C) years, body weight—63.52 kg (P) vs. 65.97 kg (C), body height—173.3 cm (P) vs. 174.21 cm (C), and BMI 20.8 (P) vs. 21.79 (C). The training period was similar in (P) and (C) groups (10.57 vs. 9.17), as was the number of practice units during the week (5.43 vs. 5.5) and number of training hours (10.57 vs. 10.67). The characteristics of cyclist groups were comparable and did not significantly differentiate them (*p* > 0.05). Supplementation intervention was used for a period of 4 months. The participants were informed not to consume nutritional supplements, yogurt, other probiotic-related products, or antibiotics during the experiment. The probiotic preparation contained strains of 13 bacteria at a concentration of over 20 billion probiotic bacteria (CFU), while potato starch was placed in a placebo capsule. The experimental group received one capsule of probiotic bacteria strains (1 × 10^11^ CFU) daily, which contained: Lactobacillus plantarum, Lactobacillus casei, Lactobacillus rhamnosus, Bifidobacterium breve, Lactobacillus acidophilus, Bifidobacterium longum, Bifidobacterium bifidum, Bifidobacterium infantis, Lactobacillus helveticus, Lactobacillus fermentum, Lactobacillus bulgaricus, Lactococcus lactis, and Streptococcus thermophilus. Placebo and probiotic capsules were identical in appearance. The energy value of the diet and the supply of individual nutrients were found to be similar in both groups and comparable throughout the studied period.

The study was performed according to the principles of the Declaration of the Helsinki World Medical Association. The research project was approved by the Bioethics Committee at the Regional Medical Chamber in Kraków No. 76/KBL/OIL/2017. All participants were informed of the possibility to withdraw at any time, engaged in the daily recording of their subjective feelings related to digestive system discomforts (nausea, vomiting, diarrhea, abdominal pain, heartburn, and gastrointestinal bleeding) with a determination of such feelings’ severity (0—none, 1—mild, 2—moderate, and 3-severe), and maintained a training diary describing the distance covered, the duration, and the intensity of the training on a scale of 1–5 (1-light effort; 5-heavy effort). Dietary monitoring scheme included keeping food diaries and the rules governing eating and the storage of probiotics/placebo. During the experiment, the participants were advised to maintain a healthy lifestyle; were prohibited from using drugs, supplements, or other probiotic agents; their consumption of products containing probiotic bacteria was limited to no more than several times a week; their consumption of caffeine and alcoholic beverages 24 h before the study was banned; they were required to eat a standardized breakfast 2 h before the capacity exercise; and were required to avoid training the day before the tests.

**Figure 1:**
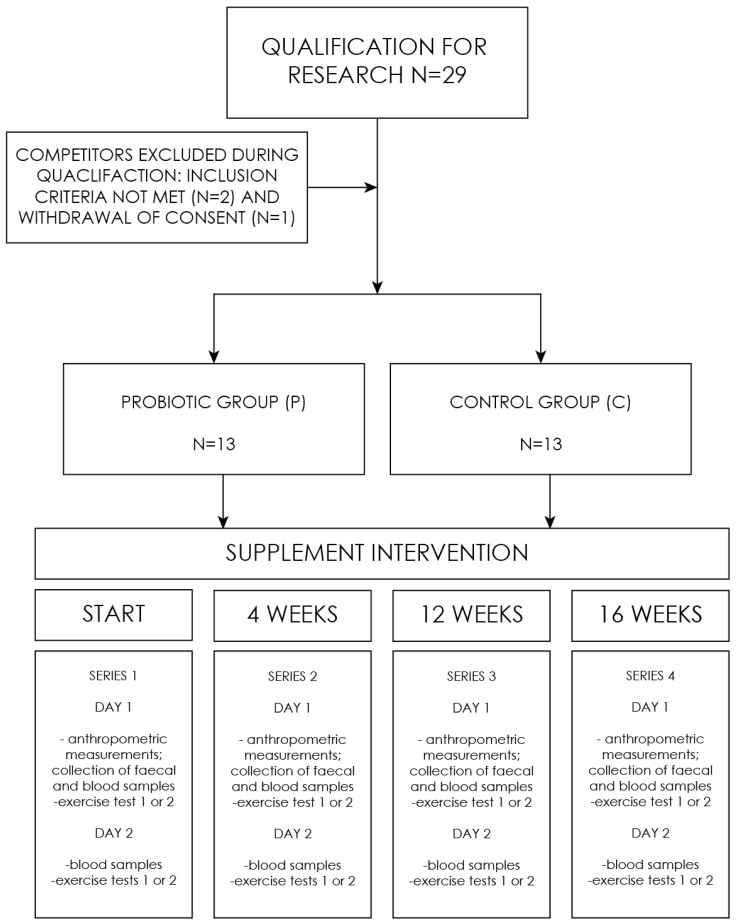
Research process flowchart.

The experiment comprised a series of studies, consisting of four (1–4) follow-up measurements: the baseline (1), after 4 weeks (2), after 12 weeks (3), and the final measurement after 16 weeks (4). On the first day, supplement capsules (probiotic/placebo) were provided to the respondents. A single measurement included determinations made on two days (Figure 1). First day included: a health assessment questionnaire, anthropometric measurements, collection of blood, and fecal sampling. In addition, on the first day of the study, one of the subgroups performed the aerobic test, and the other subgroup performed the anaerobic test. On the second day, the situation was reversed. The division of the groups was constant. All the tests were performed under the supervision of a sports medicine physician.

The level of maximal anaerobic power was assessed using a modified version of the Wingate test. The test was performed after a 5-min standard warm-up on a cycle ergometer (ER 900 D–72,475 BIT 2 Jaeger, Germany) followed by a 5-min rest period. The test consisted of a maximum 20-s effort with an individually selected load (7.5% of the body weight). The following factors were assessed: level of total work (W_glob_.) and maximal anaerobic power (MAP), average power per revolution (P), mean time to achieve maximal anaerobic power (t_o_), and time to maintain maximal anaerobic power (t_u_).

Aerobic test was carried out on a cycle ergometer (ER 900 D–72,475 BIT2 Jaeger, Friedberg, Germany) using a computerized mobile ergo-spirometer (Start 2000 M, MES, Poland) programmed for measurements at 30-s intervals. The test was started with a load of 100 W; then, every two minutes, the resistance was increased by 35 W. The pedaling rhythm was constant at 90 rpm. The trial was continued until the participant was subjectively exhausted. During the aerobic test, selected indices of the respiratory and circulatory systems were analyzed, including maximal oxygen uptake (VO_2max_), exercise duration (t), maximum power (P_max_), and maximal heart rate (HR_max_). The heart rate (HR) was monitored every 30 s during the entire exercise (Polar Electro, Kempele, Finland). The perceived physical fatigue was assessed using the Borg-20 scale every 5 min.

The anthropometric measurements assessed body height (BH), body mass (BM), fat mass (FM), and lean body mass (LBM), and were determined using the Akern Body Comp–MF + bioimpedance body composition analyzer (Italy).

The lactate levels were determined based on a blood sample collected from the capillaries of finger pulp (10 µL) 5 min before the physical effort and then 3, 10, and 20 min after the effort, and concentration was measured with a miniphotometer (Plus LP 20, DR LANGE, Germany). The blood samples were taken before each test to determine biochemical blood indices and before and after the exercise test in order to evaluate the pro-inflammatory interleukin (IL-1β, IL-6, and IL-8) and anti-inflammatory (IL-10) markers as well as the anti/pro-oxidative potential (TAS/TOS) levels of IgA and tumor necrosis factor (TNF-alpha). Stool samples were collected to determine the concentration of zonulin and alpha-1 antitrypsin.

MS EXCEL 2007 spreadsheets were used for collected results and PQStat version 1.6.8.312 (Poznan, Poland) for statistical analysis and interpretation. To compare the effect of supplementation with a multispecies probiotic preparation on the changes in the analyzed variables in the group of cyclists, 2-way analysis of variance (ANOVA) was used with repeated measures on time. The normality of the data was checked using the Shapiro–Wilk test. Comparisons of data where the distribution deviated from normal were performed using the non-parametric Mann–Whitney U test. Comparison of the results of quantitative scales between the first measurement and the fourth was performed with the Wilcoxon pairwise test. Statistical significance of differences between the compared means was assumed at *p* < 0.05, and probabilities at *p* < 0.01 were considered highly significant.

## 3. Results

### 3.1. The Level of Cyclists’ Capacity

The analysis of the data regarding the changes in the participants’ maximal oxygen uptake (VO_2 max_), exercise duration (t), maximum power (P_max_), and maximal heart rate (Hr_max_) showed that a 16-week supply of a probiotic preparation resulted in a significant increase in the participants’ maximal oxygen uptake, an increase in their exercise time to failure, and a significant decrease in maximal heart rate (Table 1).

The blood lactate concentration after four months in the 3rd, 10th, and 20th minutes after the aerobic test decreased by 7.89%, 19.96%, and 14.38%, respectively, in group P, and a significant reduction was observed in the probiotic group between the pre-testing and post-testing measurements at 10 (*p* = 0.01) and 20 min (*p* = 0.0002) (Table 2).

There were no significant statistical differences in the subjective feeling of work annoyance (fatigue) according to the Borg scale between the groups upon the follow-up measurements (Table 3). In contrast, it was observed that, after the last aerobic test, the feelings of fatigue were significantly lower (*p* = 0.0026) in the P group than in the C group.

Four months of probiotic supplementation did not affect the level of global workload, maximal anaerobic power per kilogram of body weight, magnitude of power achieved per kilogram of body weight, time to achieve maximal anaerobic power, and time to maintain maximal power of the cyclists (Table 4).

The blood lactate concentration in the 3rd, 10th, and 20th minutes after the Wingate test decreased by 9.78%, 14.95%, and 24.9%, respectively. In the P group, between the pre-testing and in the 3rd (*p* = <0.0001), 10th (*p* = 0.0001), and 20th (*p* = 0.01) minutes post-testing a statistically significant decrease in the results was observed (Table 5).

### 3.2. Analysis of Body Composition of Cyclists during the Experiment

Supplementation with multi-strain probiotics did not affect most of the body composition parameters, except for the muscle mass content, which increased slightly. There were statistically significant intergroup differences (*p* = 0.02) with respect to the muscle mass percentage. After sixteen weeks of the nutritional intervention, the probiotic supplementation group showed a significant increase in muscle mass compared to the baseline (Table 6).

### 3.3. Analysis of Selected Markers of Intestinal Membrane Permeability

**Figure 2:**
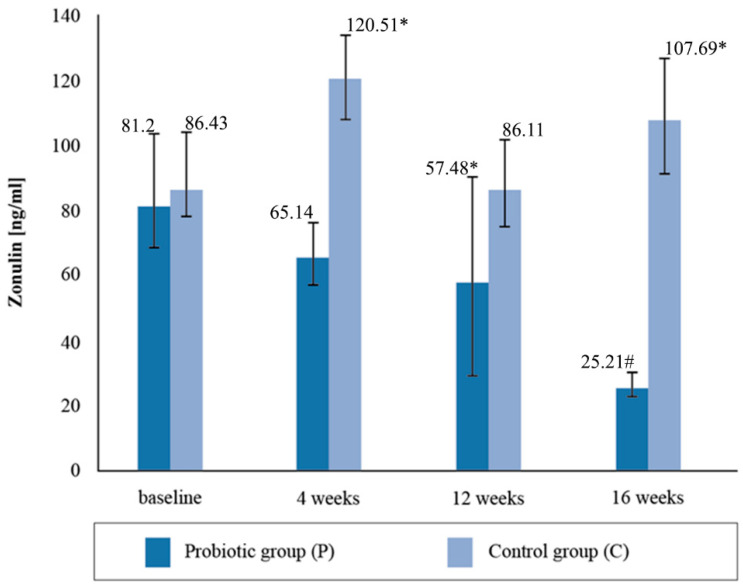
Concentration of zonulin in stool at follow-up measurements during supplementation intervention. * Statistically significant intergroup differences (*p* < 0.05); # statistically significant differences compared with the initial value (*p* < 0.05).

The mean zonulin concentration in the stool before the nutritional intervention did not statistically differentiate between the groups of cyclists, with both groups exceeding the reference values for the intestinal permeability marker and being higher than normal (Figure 2). The normal range for zonulin is <60 ng/mL. After four weeks of supplementation, the concentration of zonulin decreased by 21%, and in the placebo group it increased by 39.4%. After the twelve-week probiotic supplementation, the concentration of zonulin in the P group decreased again by 10%, and in the C group it decreased by 28%. In the placebo group, at the third determination, the mean zonulin concentration was above the reference range. After four months of supplementation, the mean concentration of the zonulin marker decreased by a further 43.87% in the group taking the probiotic preparation and increased by 25.07% in the placebo group. The 16-week probiotic supplementation significantly reduced the concentration of the intestinal permeability marker compared to the baseline (*p* = 0.0035).

**Figure 3:**
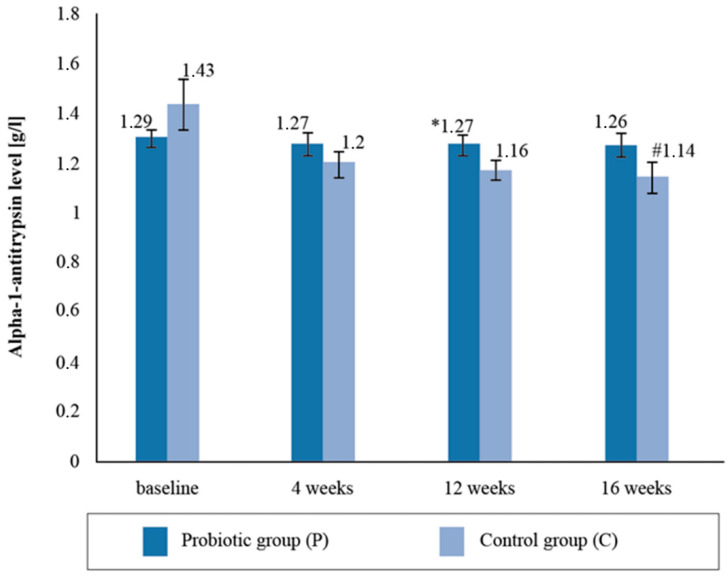
Alpha-1-antitrypsin level in the blood serum at follow-up measurements during supplementation intervention. * Statistically significant intergroup differences (*p* < 0.05) # Statistically significant differences compared with the initial value (*p* < 0.05).

There were no statistically significant intergroup differences with respect to the alpha-1- antitrypsin (AAT) levels in the blood serum upon the follow-up measurement. There was a statistically significant reduction in the mean AAT levels (by 20.2%) between the baseline and post-supplementation in the placebo group (Figure 3).

**Figure 4:**
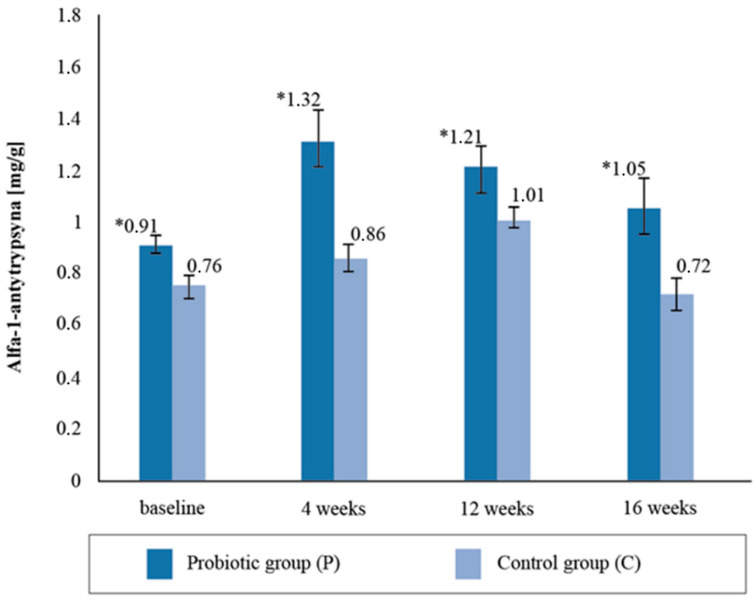
Alfa-1-antytrypsin concentration in the stool sample at follow-up measurements points during supplementation intervention. * statistically significant intergroup differences (*p* < 0.05).

Upon assessing the alpha-1- antitrypsin content in the stool samples, statistically significant intergroup differences (*p* = 0.02) were found with respect to the baseline collection; the concentration in the P group was 16.03% higher compared to the C group (Figure 4). The difference in the intergroup results after the fourth week’s stool AAT measurement was 34.09%, after twelve weeks it was 16.6%, and after sixteen weeks it was 31.13%. The AAT concentration was observed to be statistically higher in the P group than in the C group throughout the study period. There were no significant differences in the AAT concentrations between the baseline and 16 weeks post-supplementation (*p* = 0.7263).

### 3.4. Analysis of the Concentration of Immunoglobulin A and Pro-Inflammatory (TNF-α, Il-1β, Il-6, and Il-8) and Anti-Inflammatory Cytokines (Il-10)

Prior to the experiment, the IgA concentrations did not differ between the groups; similarly, after four, twelve, and sixteen weeks of supplementation, no significant changes in the immunoglobulin concentrations were found. At the end of the experiment, there was a significant increase in the IgA immunoglobulin levels in the probiotic supplementation group (*p* = 0.01) compared to the baseline values, while the IgA levels in the placebo group remained the same throughout the supplementation intervention (Table 7).

The determinations of IL-1β, IL-6, IL-8, and anti-inflammatory IL-10 levels (due to financial constraints) were performed at the baseline and 16 weeks post-supplementation. The cytokine concentrations were assessed twice: before the exercise test and 20 min after the exercise test.

The mean TNF-α concentration before the aerobic test was significantly lower in group P compared to group C. After one month of supplementation, the serum TNF-α concentration before the aerobic test increased significantly by 4.64 mL/U. After the aerobic test, it decreased by 2.49 mL/U in the group taking the probiotic preparation. The differences in the TNF-α concentrations between the P and C groups were statistically significant (*p* = 0.0001). The differences in the TNF-α concentrations between the P and C groups after 12 and 16 weeks were statistically significant, and no significant differences in TNF-α concentrations were found between the baseline and post-supplementation measurements. In the P group, the significantly lower (*p* < 0.05) concentration of the TNF-α marker between the pre- and post- supplementation periods in the aerobic test were demonstrated (Table 8).

Prior to the Wingate test, no statistically significant differences were observed between the TNF-α levels in the participants with respect to the supplementary intervention and placebo. There was also no significant effect of the probiotic supplementation after one, three, and four months on the TNF-α levels tested prior to anaerobic exercise. After the Wingate test, at the baseline, highly significant statistical differences in the tumor necrosis factor concentration were found between the groups. After sixteen weeks of supplementation with the probiotic preparation, there was a significant reduction in TNF-α levels compared to the baseline value (Table 8).

The IL-1β concentration results obtained in the blood samples of all the athletes were below 1 pg/mL and, despite performing a high-sensitivity test, the results were too low to facilitate an interpretation (Table 9).

During the aerobic test there were no statistically significant intergroup differences in the serum IL-6 levels in any of the measurements taken prior to aerobic testing (Table 10). There was a significant decrease in the IL-6 concentrations between the pre-exercise and post-exercise measurements after the Wingate test.

There were significant differences in the IL-8 levels between the groups prior to the aerobic test in the baseline values. After four months of the probiotic intervention, the difference in both groups was not statistically significant. After the Wingate test there were no differences in the mean concentration of IL-8 during the experiment (Table 11).

There were no significant differences between the groups with respect to the concentration of IL-10 before the aerobic test in the entire study (Table 12). The mean IL-10 levels in the blood serum in the Wingate test were significantly higher in the P group than in the C group. A statistically significant reduction in the IL-10 levels was observed in the P group between the pre-exercise and post-exercise samplings. Prior to the studies in the group with the probiotic supplementation, the mean level of IL-10 after the Wingate test was statistically significant.

### 3.5. Analysis of the Pro-Oxidative and Antioxidant Potential of Cyclists’ Blood

No effect of the multi-strain probiotic preparation was observed on the total oxidative status (TOS) and total antioxidant status (TAS) of the blood plasma measured after the aerobic test (Table 13 and Table 14). There were significant intergroup differences in the levels of the total oxidative status (TOS) examined before the Wingate exercise test. After four weeks of supplementation, the TOSs in both groups decreased significantly: in the P group to 497.7 ± 266.7 μmol/l, and in the group to 711.2 ± 280 μmol/l. After twelve weeks of the supplementation intervention, the level of the TOS increased significantly in the P group by 7%, and in the placebo group it decreased by 8%. The level of the total plasma oxidative status after four months of the nutritional intervention decreased by 10% in the P group and increased by 40% in the C group. There was a statistically significant decrease in the TOS level in the P group post-supplementation in relation to the baseline values (Table 13).

The TOS levels after the Wingate test were significantly higher in group P throughout the experiment. The P group showed a statistically significant reduction in TOS levels post-supplementation in relation to the baseline values.

## 4. Discussion

Possessing a high aerobic capacity in road cycling, as an endurance sport, allows the athletes to achieve long-term exercise, delays the onset of anaerobic changes, and determines the efficiency of post-exercise recovery and the rate of regeneration of the body, in turn determining the athlete’s ability to compete effectively [39]. The positive effect of probiotics on physical capacity, extending athletes’ stamina during an aerobic test and increasing their VO_2max_, was shown in the reports of O’Brien [40], Shing [42], and Salarkia et al. [33] in runners and swimmers. Similar results were observed in our research, in which, after a 4-month probiotic supplementation, the values of the VO_2max_ increased significantly by 5.98%, while in the placebo group they were slightly decreased (by 1.72%). In contrast, Marshall [43] and Lamprecht et al. [44] did not observe significant differences with respect to the VO_2max_ levels between a group of athletes taking a probiotic and a placebo. The test results differ in protocol, i.e., the composition of the probiotics, the dose, the duration of the test, the evaluation of the results, and the population. The potential benefits of the probiotic supplementation towards exercise capacity are also unclear. There might be several explanations for how the probiotic supplementation improved the aerobic capacity of the athletes. Microbiota in the gut may play a role in the body’s energy system after a few minutes of muscle contraction, when the phosphocreatine concentration decreases, resulting in the need for other fuels. Gene expression for glycogenolysis would be induced to ensure ATP production for increased muscle activity requirements such as the cross-bridge cycle, myosin ATPase activity, and muscle ion pumps [45]. Most studies suggest that well-trained individuals achieve higher blood lactate concentrations during maximal intensity exercise, reflecting a specific adaptation to exercise and the ability to continue. In the present study, sixteen weeks of supplementation with a multi-strain probiotic preparation resulted in a reduction in the post-exercise LA concentrations occurring after an aerobic test in the 3rd, 10th, and 20th minutes by 9.98%, 19.96%, and 14.38%, respectively. Therefore, it can be assumed that probiotic supplementation shortened the time of post-exercise restitution. In Shing’s research [38], it was shown that supplementation with a multi-strain probiotic preparation increased the exercise time (37 min vs. 33 min) as in our study, where the duration of exercise increased (by 9.05% for the probiotic vs. 4.8% for the placebo). The benefits of probiotic supplementation with respect to exercise capacity are also unclear. Some studies have shown conflicting results [42,43], while others have shown significant intergroup differences [41,45] or their absence [38,42]. The relevant participants’ increase in their VO_2max_ and ergometer load, reduction in heart rate, extension of the time of effort, and reduction in post-exercise LA concentration can be considered the beneficial effects of the probiotic therapy on the aerobic physical capacity of the cyclists. It should be noted that the present study involved professional road cyclists, maintaining a training regime both before and throughout the study. Therefore, even a slight increase in aerobic capacity can be considered a very beneficial phenomenon. It was additionally observed that, following aerobic exercise in the group taking the probiotic for 16-weeks, the athletes felt less discomfort performing the test.

In competitions where athletes perform at maximal and supramaximal intensities, an efficient energy supply via anaerobic metabolism is required [46,47,48,49,50,51]. Such competitions include road cycling, in which fast, dynamic, and effective acceleration requires the generation of very high muscle power in a short time [52]. According to a study by Jäger et al. [2], the muscle power slightly increased in the study’s probiotic group while in the placebo it decreased during the Wingate test. The use of a probiotic also reduced the degree of muscle damage and prevented a decrease in peak power. In contrast, the study by Ibrahim [46] evaluated the effect of a probiotic preparation on muscle strength and peak strength, where no significant changes between a group with probiotic supplementation and a placebo group were found, which is consistent with the results obtained in this work. There were no significant differences between the probiotic group and the placebo group with respect to the level of maximal anaerobic power, the magnitude of power, and the time to reach and maintain MAP during the Wingate test. Similarly, the supplementation with a multi-strain probiotic preparation in the present study did not result in significant differences in blood lactate levels after the Wingate test.

Zonulin is considered to be a physiological modulator of intercellular connections and a marker of intestinal permeability [53,54,55,56]. Excluding the effect of dietary probiotics on the changes in the zonulin concentrations, probiotic supplementation was shown to reduce the concentration of zonulin in the stool of professional cyclists. It should be emphasized that at the beginning of the study, the zonulin levels were above normal (>60 ng/mL), and after 12 weeks of supplementation with a multispecies probiotic preparation they were within the reference ranges. The results show that the athletes had experienced intestinal permeability prior to the study, perhaps as a result of regular prolonged training, and that 16 weeks of probiotic supplementation improved the integrity of the intestinal barrier. The results of our research correspond to those obtained by Lamprecht et al. [44], in which the concentration of zonulin in stool mass significantly decreased after 14 weeks of probiotic supplementation. Furthermore, in our study, after 4 months of supplementation, significant differences were observed between the groups with respect to the concentration of AAT in the stool masses, with higher values in the P group. Meanwhile, no statistical differences were found between the groups regarding the concentration of alpha-1-antitrypsin in response to the fourteen-week probiotic supplementation in Lamprecht et al.’s study [44]. In a clinical study by Viljanen et al. [56], intestinal inflammatory markers’ AAT in stools decreased significantly when *Lactobacillus rhamnosus GG (LGG*) bacteria were used, which was not observed in the placebo group. When summarizing the data analysis of the changes in the excretory system indices and intestinal membrane permeability markers, it was shown that four months of probiotic supplementation resulted in a decrease in the concentration of zonulin in the stool mass and resulted in the maintenance of AAT concentrations at similar levels during the experiment.

Based on the analysis of the data on the changes in IgA levels and pro-inflammatory (Il-1β, Il-6, and Il-8) and anti-inflammatory (Il-10) cytokine levels, it was shown that four months of probiotic supplementation resulted in an increase in IgA and a decrease in tumor necrosis factor TNF-α measured after aerobic and anaerobic testing, in IL-6 measured before and after anaerobic testing, and in mean IL-10 measured before anaerobic testing. No effect of probiotic supplementation on the mean concentrations of Il-1β and Il-8 was found. Supplementation with probiotic strains not only contributes to the elimination of dysbiosis, but also has a beneficial effect on immunity, reducing the need for antibiotics and the frequency of infections of the upper respiratory tract and gastrointestinal tract, which is particularly important for athletes [14,57,58,59]. As a consequence of the activation of the relevant signaling pathways, the production and secretion of pro inflammatory cytokines such as TNF-α occurs along with interleukins IL-1, IL-6, IL-8, and IL-12 [57,58,59]. However, studies evaluating the mechanisms of action of probiotics note divergent results. The effects of interleukin-6, 8, and 10 on TNF-α are controversial [59,60,61]. There are studies that observed no beneficial effect of probiotic therapy on immunity [60,61,62,63,64], except for the study by Gleeson et al. [13], which showed an increase in immunoglobulin A in the experimental group. Unfortunately, studies assessing changes in inflammatory markers often have different protocols primarily related to varying lengths of supplementation, the different strains used, and the diverse populations of individuals that are included, making comparisons difficult. In Lamprecht et al.’s [44] study, the group of athletes showed that the concentration of TNF-α was lower in the probiotic supplement group than in the placebo group. The concentration of IL-6 in the blood serum significantly increased at the end of the study; however, no significant effect of supplementation on the concentration of IL-6 was found. In our own research, after sixteen weeks of supplementation with a probiotic preparation, a decrease in the mean concentration of IL-6 was observed, contrary to Lamprecht et al.’s study [44]. There was also a decrease in the tumor necrosis factor TNF-α concentration after the Wingate test, similar to the above-cited studies. Shing [42] and Glesson’s [13] studies evaluating the effect of multi-strain probiotic supplementation on IL-6 and IL-10 concentrations showed that IL-6 and IL -10 concentrations increased after an exercise test (without statistical significance). Our own study also found an increase in the IL- 6 concentrations in the blood serum in the athletes after sixteen weeks of supplementation compared to the baseline concentrations, while contrary to Shing’s study [42], there was a decrease in the mean IL-10 concentration after the aerobic test compared to the initial intake. The production of cytokines in Glesson’s study was higher initially than after 8 and 16 weeks (with respect to IL-2, IL-4, IL-6, IL-8, and TNF-α), and the concentration of (IL-1β) decreased only at the 16th week. Our study, similar to the study conducted by Glesson [13], noted a statistically significant increase in intellectukin-6 and interleukin-8 concentrations compared to the baseline and, contrary to the aforementioned study, a decrease in IL-10 and TNFα concentrations after 16 weeks of supplementation with a multi-strain probiotic preparation. In the cyclists’ blood plasma, the IL-1β concentrations were below the detection standard throughout the experiment. Brand [65] and Heddle [66] presented analogous results with respect to healthy people, reporting a very low concentration of this interleukin. Marinkovic et al. [47] and Ibrahim [46] in their studies did not notice an effect of probiotic supplementation on the IL-10 level. A study by Jäger [1] showed that a three-week supplementation with a probiotic preparation (Streptococcus thermophilus FP4 and Bifidobacterium breve BR03) causes a lowering of the Il-6 concentration. The decrease in the resting IL-6 levels found in our study may suggest a general decrease in inflammation. It should also be noted, however, that all the study participants were healthy and had baseline IL-6 values within the normal range. Nevertheless, the observed change warrants further research, especially in people whose IL-6 is elevated. In our study, the mean IL-6 concentration after aerobic exercise increased in the probiotic group, which may be due to the type of exercise test, as there was a decrease in the IL-6 concentration after the Wingate test. A variable training intensity and exercise duration may have a significant effect on IL-6 concentration after exercise [67,68,69]. It was found that supplementation with a probiotic drink containing *L. casei shirota* caused an increase in sIgA concentration in athletes [13,70,71,72]. In studies by Cox et al. [58] and Tiollier et al. [72], no significant changes in IgA concentration were observed in response to probiotic supplementation. In our own research, after 16 weeks of supplementation with a multi-strain probiotic preparation, a significant increase in immunoglobulin A was noted. It was also noted that IgA levels increased steadily in the probiotic intervention group and remained the same in the placebo group throughout the study. Perhaps the increase in the IgA concentration under the influence of supplementation with a multi-strain probiotic preparation would result in a positive health effect of ‘suppressing’ inflammation. Four months of probiotic supplementation resulted in an increase in IgA and a decrease in TNF-α measured after the aerobic and anaerobic tests, in IL-6 measured before and after the anaerobic test, and in mean IL-10 measured before the anaerobic test. Probiotic supplementation has not been shown to affect the concentration of Il-1β and Il-8.

An analysis of the data concerning the changes in the pro-oxidant and anti-oxidant statuses revealed a decrease in the total oxidant status (TOS) of plasma before and after the anaerobic test at the end of the experiment in relation to the initial values. There was no effect of the probiotic supplementation on the total antioxidant status (TAS). Increased physical effort, both recreational and with the intention of participating in sports competitions, causes disturbances in the pro-oxidant–anti-oxidant balance, leading to a decrease in the plasma antioxidant status [73,74,75]. The reduction in the body’s antioxidant capacity leads to the activation of adaptive processes in the tissues, primarily aimed at protecting the cells from further stimuli causing an increase in reactive oxygen and nitrogen species [75]. After a temporary reduction in their concentration due to effort, an increase in their number above the initial values is observed during the restitution period [76]. In turn, the repeated exposure of the body to increased radio-frequency thermal ablation (RFTA) production through regular training results in an increase in the antioxidant defenses of the entire body [77]. The extent to which the body reacts to the effort through oxidative changes is determined by the intensity and type of effort. Research shows that exercise with a high proportion of eccentric contractions causes a deeper and longer lasting redox disturbance, due in part to the greater degree of muscle damage than during efforts with a predominance of concentric contractions [78,79]. It follows that the type of training used (endurance or strength) determines the degree of oxidative stress and the associated adaptive response of the athlete’s body. The research presented herein showed a slightly lower level of the total antioxidant status of the TAS in the group that received the probiotic supplementation before the start of the exercise test with an increasing load than in the group taking the placebo. The TAS levels remained similar throughout the sixteen-week study in the supplement intervention group and were reduced by 10% in the placebo consumption group compared to the first intake. This difference was not statistically significant, but a depletion of low molecular weight antioxidants in the control group was observed. In our own study, no effect of a multi-strain probiotic preparation was observed on the total antioxidant status (TAS) of the plasma measured after an aerobic test. The TAS levels in the probiotic group decreased by 10% in the last study compared to the first, but this change was not statistically significant. In analogous measurements during the anaerobic tests, there were no effects of probiotic supplementation on the level of the total antioxidant capacity (TAS) of the plasma examined before the “Wingate” exercise test. Throughout the study period, the TAS scores were lower in the supplementation intervention group compared to the placebo group. In addition, no effects of probiotic supplementation on the level of total anti-oxidant capacity (TAS) of the blood plasma following the end of the Wingate test were noticed. Throughout the study period, the TAS scores were similar between the groups. Martarelli et al. [41] showed that probiotic supplementation increased the level of anti-oxidants in the blood plasma of athletes, thus neutralizing reactive oxygen species. Similarly, in our own study, after 4 weeks of using a probiotic preparation, before and after the aerobic test, an increase in low molecular antioxidant levels was observed; on the other hand, a decrease in the antioxidant levels before and after anaerobic exercise was observed. After four weeks of the supplementation intervention, an imbalance in the pro-oxidative and anti-oxidant balance was noticed. Scientists are looking for solutions to reduce the level of oxidative stress and, consequently, reduce the occurrence of related diseases. In the studies cited by Martarella [41] it was noted that the group supplemented with a probiotic preparation showed a greater resistance to oxidative stress. According to the results, intense exercise induced oxidative stress and probiotic supplementation increased plasma antioxidant levels, thus neutralizing reactive oxygen species. Athletes and people exposed to oxidative stress can take advantage of the properties of these probiotic strains to increase their antioxidant levels and neutralize the effects of reactive oxygen species [41]. Lamprecht et al.’s studies [44], in which athletes participated, did not find an effect of probiotic strains on the level of the TOS. Our own research is consistent with the results of Lamprecht et al. [44] regarding the level of TOS during aerobic exercise. The effect of multi-strain probiotics on the oxidative status in the group with the probiotic supplementation was not noticed. Another effect was seen during the anaerobic test. In the placebo group, the level of the TOS after 4 weeks was twice as high as the initial value. After 16 weeks, decreased plasma TOS levels were noted in the multi-strain probiotic supplement group before and after the Wingate test. The statistical analysis showed that this difference was statistically significant. The results indicated that the anaerobic test induced oxidative stress and that probiotic supplementation increased plasma antioxidant levels, reducing oxidative stress.

Athletes are often more prone to various infections after strenuous training due to a temporary reduction in immunity. Probiotics could be used as supplements to improve health, but the research results in this area are inconclusive. Respiratory and gastrointestinal symptoms improve after probiotic supplementation, but not all studies support this [61]. The study results differ in protocol, namely, with respect to the probiotic composition, dose, study time, outcome assessment, and population.

## 5. Conclusions

1. The probiotic supplementation resulted in increased levels of aerobic capacity, assessed by an increase in the relative magnitude of maximal oxygen uptake, an increase in the duration of exercise to failure, an increase in the load on the ergometer, a decrease in the participants’ heart rates, and a feeling of less discomfort during the exercise test, confirming the beneficial effect of probiotics on the cyclists’ exercise capacity.
2. There were no effects of probiotic supplementation on the levels of maximal aerobic power, maximal anaerobic power per kilogram of body weight, power per revolution, and the time to achieve and maintain MAP.
3. With respect to athletes training in competitive road cycling, supplementation with multi-strain probiotics did not affect most body composition parameters, except for muscle mass content, which increased slightly.
4. As a result of the undertaken probiotic therapy, the frequency and intensity of the cyclists’ gastrointestinal complaints decreased, the concentration of zonulin in the stool mass decreased, and α1 antitrypsin was maintained at a similar level during the experiment, indicating a sealing of the intestinal barrier and beneficial changes in the cyclists’ intestinal function.
5. The probiotic supplementation resulted in a reduction in the concentrations of tumor necrosis factor TNF-α measured after the aerobic and anaerobic tests, IL-6 before and after the anaerobic test, IL-10 before the anaerobic test, total oxidative status (TOS) of the blood plasma before and after the anaerobic test, and the maintenance of IgA immunoglobulin levels throughout the experiment, as evidenced by the beneficial effect of the probiotic supplementation on the pro-oxidative and antioxidant status of the participants and their levels of immunity.
6. Due to the observed positive effects of probiotics with respect to reducing inflammation, sealing the intestinal barrier, increasing aerobic capacity, decreasing lactate concentration post-exercise, and alleviating the feeling of heaviness in all the exercise tests, it is justified to recommend probiotic supplementation to cyclists and to pay attention to the consumption of food products that are a source of probiotics.

The impact of probiotics on the athletic population is a new research area, where a limited amount of such research has been conducted to date, and although this research has shown great promise, little is known about the benefits of probiotics in highly active individuals and, ultimately, whether they benefit from them. Future investigations could consider unifying methodological approaches and evaluating the physiological effects of probiotics.

## 6. Limitations

The first limitation concerns the criteria for inclusion in the studied group, which were experience in competitive cycling for at least 5 years, a male sex, an age between 18–26 years, and a high sporting level (participation in national and international cycling competitions), yielding only 26 participants. This research did not evaluate the intestinal microbiota of the athletes. The modifications in the intestinal epithelium and the composition of intestinal microbiota could not be examined.

## Figures and Tables

**Table 1:** Maximal oxygen uptake (VO_2 max_), exercise duration (t), maximum power (P_max_), and maximal heart rate (HR_max_) during the aerobic test at follow-up measurements during supplementation intervention.

Indicator/Measurement		Probiotic Group (P)			Control Group (C)			*p*
		x	SD	SE	x	SD	SE	
VO_2max_ [mL/kg/min]	baseline	65.28 *	6.00	1.66	58.03 *	0.52	2.92	*p* = 0.1336
	4-weeks	63.62 *	5.79	1.60	58.45 *	7.99	2.22	*p* = 0.0069
	12-weeks	65.03	5.77	1.60	60.36 *	8.38	2.33	*p* = 0.45
	16-weeks	69.18 *^#^	5.69	1.58	**57.03**	6.69	1.86	*p* = 0.006
t [min]	baseline	14.35	1.80	0.50	14.92	1.12	0.31	*p* = 1.0000
	4-weeks	15.00	1.15	0.32	14.88	1.75	0.48	*p* = 0.9483
	12-weeks	15.12	2.11	0.58	15.04	2.14	0.59	*p* = 0.7
	16-weeks	**15.65 ^#^**	2.02	0.56	**15.23**	1.89	0.53	*p* = 1.0000
P_max_ [W/kg]	baseline	5.11	0.68	0.19	4.83	0.71	0.20	*p* = 0.3531
	4-weeks	5.29	0.47	0.13	4.88	0.81	0.23	*p* = 0.70
	12-weeks	5.38	0.68	0.19	4.91	0.85	0.24	*p* = 0.9323
	16-weeks	5.36	0.60	0.17	4.86	0.79	0.22	*p* = 0.7595
HR_max_ [bpm]	baseline	193.3 *	3.01	0.84	182.8 *	7.74	2.15	*p* = 0.0031
	4-weeks	193.8 *	4.24	1.18	182.8 *	7.69	2.13	*p* = 0.003
	12-weeks	192.0 *	6.76	1.87	184.0 *	0.47	2.90	*p* = 0.003
	16-weeks	188.6 *^#^	7.08	1.96	182.9 *	9.36	2.60	*p* = 0.01

x—mean; SD—standard deviation; SE—standard error; * Statistically significant intergroup differences (*p* < 0.05); ^#^ Statistically significant differences compared with the baseline (*p* < 0.05).

**Table 2:** The blood lactate concentrations at baseline, 3, 10, and 20 min post-aerobic testing at follow-up measurements during supplementation intervention.

Indicator/Measurement		Probiotic Group (P)			Control Group (C)		
		x	SD	SE	x	SD	SE
LA [mmol/L] pre-exercise	baseline	2.33	0.48	0.13	1.78	0.38	0.11
	4-weeks	3.09	1.80	0.50	2.14	0.39	0.11
	12-weeks	2.71	1.80	0.50	1.84	0.24	0.07
	16-weeks	2.34	0.87	0.24	2.57	0.64	0.18
LA [mmol/L] 3 min post-exercise	baseline	13.42	1.39	0.38	12.38	2.50	0.69
	4-weeks	14.36	1.97	0.55	13.44	3.44	0.95
	12-weeks	12.39	2.51	0.70	12.95	3.03	0.84
	16-weeks	12.36	2.94	0.81	12.24	2.61	0.72
LA [mmol/L] 10 min post-exercise	baseline	11.12	1.25	0.35	10.26	2.74	0.76
	4-weeks	11.67	1.74	0.48	10.03	2.65	0.73
	12-weeks	9.95	2.87	0.80	9.48	3.49	0.97
	16-weeks	**8.90 ^#^**	2.76	0.76	10.22	2.86	0.79
LA [mmol/L] 20 min post-exercise	baseline	7.16	1.53	0.43	6.53	2.54	0.70
	4-weeks	8.55	2.15	0.60	7.11	2.22	0.62
	12-weeks	6.14	1.85	0.51	6.02	2.86	0.79
	16-weeks	**6.13 ^#^**	2.16	0.60	6.82	2.43	0.67

x—mean; SD—standard deviation; SE—standard error; ^#^ Statistically significant differences compared with the baseline (*p* < 0.05).

**Table 3:** The subjective feeling of fatigue according to the Borg scale during the aerobic test.

Measurement	Probiotic Group (P)			Control Group (C)			*p*
	x	SD	SE	x	SD	SE	
baseline	19.38	0.48	0.13	19.30	0.72	0.20	*p* = 0.9542
4-weeks	19.30	0.6	0.16	19.53	0.49	0.13	*p* = 0.3674
12-weeks	19.00	0.55	0.15	19.07	0.88	0.22	*p* = 0.78
16-weeks	18.43 *	0.6	0.16	19.38 *	0.73	0.20	*p* = 0.0026

x—mean; SD—standard deviation; SE—standard error; * Statistically significant intergroup differences (*p* < 0.05).

**Table 4:** Global workload W_glob_. [kJ], level of maximal anaerobic power MAP [W], relative-to-body-weight average anaerobic power MAP [W∙kg^−1^], average power P [W], relative-to-body-weight average power P [W∙g^−1^], mean time to achieve maximal anaerobic power t_o_ [s], and time to maintain maximal anaerobic power t_u_ [s] at follow-up measurements during supplementation intervention.

Indicator/Measurement		Probiotic Group (P)			Control Group (C)			*p*
		x	SD	SE	x	SD	SE	
W_glob_. [kJ]	baseline	12.59	1.18	0.33	13.84	1.49	0.41	*p* = 0.1
	4-weeks	12.69	1.10	0.31	13.91	1.50	0.42	*p* = 0.08
	12-weeks	12.78	0.98	0.27	13.63	1.74	0.48	*p* = 0.82
	16-weeks	12.83	0.07	0.30	13.64	1.69	0.47	*p* = 0.94
MAP [W]	baseline	742.7	72.31	2 0.05	816.6	99.44	27.58	*p* = 0.07
	4-weeks	753.0	69.46	19.27	817.6	102.8	28.51	*p* = 0.22
	12-weeks	746.1	61.83	17.15	791.6	108.5	30.08	*p* = 0.71
	16-weeks	742.0	65.52	18.17	801.1	115.4	22.01	*p* = 0.94
MAP [W·kg^−1^]	baseline	11.69	1.09	0.30	11.67	2.56	0.10	*p* = 0.94
	4-weeks	11.71	1.05	0.2	11.61	0.5	0.24	*p* = 0.472
	12-weeks	11.60	0.79	0.22	11.77	2.60	0.31	*p* = 0.472
	16-weeks	11.66	0.76	0.21	11.84	2.61	0.29	*p* = 0.474
P [W]	baseline	629.5	58.96	16.35	692.1	74.44	20.65	*p* = 0.06
	4-weeks	634.4	55.40	15.37	695.6	75.07	20.82	*p* = 0.08
	12-weeks	638.7	48.90	13.56	680.4	85.50	23.71	*p* = 0.06
	16-weeks	641.5	53.66	14.88	682.5	85.97	23.85	*p* = 0.655
P [W·kg^−^^1^]	baseline	9.92	0.93	0.26	9.75	0.46	0.13	*p* = 0.4
	4-weeks	9.87	0.85	0.23	9.89	0.51	0.14	*p* = 0.06
	12-weeks	9.93	0.63	0.17	9.55	0.69	0.19	*p* = 0.26
	16-weeks	10.00	0.66	0.18	9.66	0.63	0.17	*p* = 0.261
t_o_ [s]	baseline	4.26	0.76	0.21	3.83	0.48	0.13	*p* = 0.31
	4-weeks	3.94	0.60	0.17	3.78	0.25	0.07	*p* = 0.062
	12-weeks	3.91	0.45	0.13	3.59	0.33	0.09	*p* = 0.472
	16-weeks	4.01	0.58	0.16	3.67	0.27	0.08	*p* = 0.65
t_u_ [s]	baseline	3.39	0.87	0.24	3.31	0.43	0.12	*p* = 0.7
	4-weeks	3.16	0.67	0.19	3.16	0.30	0.08	*p* = 1.00
	12-weeks	3.18	0.52	0.14	3.36	0.46	0.13	*p* = 0.089
	16-weeks	3.42	0.45	0.12	3.04	0.67	0.19	*p* = 0.08

x—mean; SD—standard deviation; SE—standard error.

**Table 5:** The blood lactate concentrations at baseline, 3, 10, and 20 min post-exercise for the anaerobic test at follow-up measurements during supplementation intervention.

Indicator/Measurement		Probiotic Group (P)			Control Group (C)		
		x	SD	SE	x	SD	SE
LA [mmol/L] pre-exercise	baseline	2.18	0.31	0.09	1.92	0.52	0.14
	4-weeks	2.06	0.41	0.11	2.09	0.53	0.15
	12-weeks	2.13	0.49	0.13	2.12	0.44	0.12
	16-weeks	1.76	0.44	0.12	2.03	0.43	0.12
LA [mmol/L] 3 min post-exercise	baseline	11.14	1.86	0.52	11.59	1.98	0.55
	4-weeks	13.16	2.11	0.59	11.93	3.01	0.83
	12-weeks	10.43	1.63	0.45	10.21	2.26	0.63
	16-weeks	**10.05 ^#^**	0.87	0.24	11.52	2.28	0.63
LA [mmol/L] 10 min post-exercise	baseline	11.23	1.88	0.52	11.71	2.46	0.68
	4-weeks	11.51	1.81	0.50	10.45	3.32	0.92
	12-weeks	9.34	1.50	0.42	8.76	2.10	0.58
	16-weeks	**9.55 ^#^**	1.63	0.45	9.30	2.09	0.58
LA [mmol/L] 20 min post-exercise t	baseline	7.55	1.85	0.51	8.26	1.60	0.44
	4-weeks	7.16	2.09	0.58	7.80	2.17	0.60
	12-weeks	6.60	1.25	0.35	7.45	2.13	0.59
	16-weeks	**5.67 ^#^**	1.22	0.34	7.65	1.91	0.53

x—mean; SD–standard deviation; SE—standard error; ^#^ Statistically significant differences compared with the baseline (*p* < 0.05).

**Table 6:** Body weight, BMI, fat-free mass (FFM), fat mass (FM), and muscle mass (MM) at follow-up measurements during supplementation intervention.

Indicator/Measurement		Probiotic Group (P)			Control Group (C)		
		x	SD	SE	x	SD	SE
Body weight [kg]	baseline	63.52	3.77	1.05	65.17	8.44	2.34
	4-weeks	63.97	4.26	1.18	64.14	7.55	2.09
	12-weeks	64.14	4.41	1.22	65.92	7.35	2.04
	16-weeks	63.92	4.15	1.15	66.52	7.45	2.07
BMI [kg/m^2^]	baseline	20.80	1.11	0.31	21.79	2.04	0.57
	4-weeks	20.95	1.33	0.37	21.18	1.90	0.53
	12-weeks	20.92	1.20	0.33	21.77	1.47	0.41
	16-weeks	21.06	1.03	0.29	21.97	1.26	0.35
FFM [%]	baseline	92.15	2.60	0.72	90.59	5.17	1.43
	4-weeks	92.63	3.75	1.04	91.00	5.86	1.63
	12-weeks	93.00	13.33	3.70	89.60	6.47	1.79
	16-weeks	96.10	3.27	0.91	90.11	6.12	1.70
FM [%]	baseline	7.85	2.60	0.72	9.41	5.17	1.43
	4-weeks	7.37	3.75	1.04	9.00	5.97	1.66
	12-weeks	7.00	3.95	1.10	9.40	6.47	1.79
	16-weeks	5.9	2.82	0.78	9.89	6.12	1.70
MM [%]	baseline	51.95 *	2.28	0.63	47.78 *	8.66	2.40
	4-weeks	53.40 *	2.82	0.78	48.95 *	9.33	2.59
	12-weeks	55.00 *	4.06	1.13	53.09 *	4.39	1.22
	16-weeks	58.68 *^#^	6.19	1.72	52.83 *	4.02	1.12

x—mean; SD—standard deviation; SE—standard error. * statistically significant intergroup differences (*p* < 0.05); ^#^ statistically significant differences compared with the initial value (*p* < 0.05).

**Table 7:** Immunoglobulin A (IgA) concentration in blood serum at follow-up measurements during supplementation intervention.

Indicator/Measurement		Probiotic Group (P)			Control Group (C)			*p*
		x	SD	SE	x	SD	SE	
Ig A [mg/mL]	baseline	1.85	1.13	0.31	1.59	0.59	0.16	*p* = 0.31
	4-weeks	1.88	1.01	0.28	1.58	0.58	0.16	*p* = 0.38
	12-weeks	1.95	1.00	0.28	1.58	0.60	0.17	*p* = 0.3583
	16-weeks	2.03 ^#^	0.98	0.27	1.61	0.60	0.17	*p* = 0.9539

x—mean; SD—standard deviation; SE—standard error; ^#^ Statistically significant differences compared with the baseline (*p* < 0.05).

**Table 8:** The mean TNF-α values during pre- and post-aerobic and during pre- and post- anaerobic tests at follow-up measurements during supplementation intervention.

Indicator/Measurement		Probiotic Group (P)			Control Group (C)			*p*
		x	SD	SE	x	SD	SE	
TNF-α before aerobic test	baseline	9.65 *	5.84	1.62	14.26 *	5.32	1.47	*p* = 0.03
	4-weeks	14.29 *	7.71	2.14	20.59 *	5.60	1.55	*p* = 0.0001
	12-weeks	9.54 *	4.08	1.13	15.26 *	7.58	2.10	*p* = 0.0204
	16-weeks	10.15 *	2.76	0.77	11.72 *	8.11	2.25	*p* = 0.0471
TNF-α after aerobic test	baseline	13.88 *	5.90	1.64	11.28 *	4.21	1.17	*p* = 0.0018
	4-weeks	11.39 *^#^	6.01	1.67	25.32 *	10.51	2.92	*p* = 0.0001
	12-weeks	9.75 *^#^	3.89	1.08	20.62 *	10.96	3.04	*p* = 0.0175
	16-weeks	9.84 *^#^	4.30	1.19	14.74 *	10.36	2.87	*p* = 0.031
TNF-α before anaerobic test	baseline	7.34	3.05	0.85	13.49	8.65	2.40	*p* = 0.2719
	4-weeks	10.71	5.08	1.41	10.35	1.91	0.53	*p* = 0.9326
	12-weeks	8.85	4.12	1.14	10.55	2.81	0.78	*p* = 0.7893
	16-weeks	9.42	5.09	1.41	9.32	3.43	0.95	*p* = 0.742
TNF-α after anaerobic test	baseline	8.54 *	6.37	1.77	21.71 *	7.26	2.01	*p* = 0.0035
	4-weeks	10.48 *	6.77	1.88	16.81 *	8.96	2.48	*p* = 0.0445
	12-weeks	7.78 *^#^	2.89	0.80	14.74 *	9.97	2.76	*p* = 0.0282
	16-weeks	6.8 *^#^	3.41	0.95	13.53 *	11.02	3.06	*p* = 0.0204

x—mean; SD—standard deviation; SE—standard error; * Statistically significant intergroup differences (*p* < 0.05) ^#^ Statistically significant differences compared with the initial value (*p* < 0.05).

**Table 9:** The concentration of 1β (IL-1 β) pre- and post-aerobic tests and pre- and post- anaerobic test at follow-up measurements during supplementation intervention.

Indicator/Measurement		Probiotic Group (P)			Control Group (C)		
		x	SD	SE	x	SD	SE
IL-1β [pg/mL] before aerobic test	baseline	0.065	0.041	0.022	0.109	0.021	0.041
	16 weeks	0.094	0.056	0.033	0.103	0.060	0.039
IL-1β [pg/mL] after aerobic test	baseline	0.084	0.024	0.029	0.083	0.052	0.031
	16 weeks	0.110	0.064	0.038	0.082	0.228	0.031
IL-1β [pg/mL] before anaerobic test	baseline	0.107	0.051	0.037	0.066	0.030	0.025
	16 weeks	0.094	0.056	0.033	0.075	0.015	0.028
IL-1β [pg/mL] after anaerobic test	baseline	0.097	0.029	0.034	0.071	0.002	0.026
	16 weeks	0.110	0.064	0.038	0.099	0.060	0.037

x—mean; SD—standard deviation; SE—standard error.

**Table 10:** The concentration of Interleukin 6 (IL-6) pre- and post-aerobic test and pre- and post-anaerobic test at follow-up measurements during supplementation intervention.

Indicator/Measurement		Probiotic Group (P)			Control Group (C)			*p*
		x	SD	SE	x	SD	SE	
IL-6 [pg/mL] before aerobic test	baseline	1.18	0.41	0.11	1.09	0.17	0.05	*p* = 0.11
	16-weeks	0.80	0.27	0.08	1.23	0.46	0.13	*p* = 0.0801
IL-6 [pg/mL] after aerobic test	baseline	1.71	0.72	0.20	1.14	0.18	0.05	*p* = 0.123
	16-weeks	1.76	1.00	0.28	1.65	0.24	0.07	*p* = 0.4227
IL-6 [pg/mL] before anaerobic test	baseline	1.20 *	0.37	0.10	2.61 *	2.26	0.63	*p* = 0.0272
	16-weeks	0.86 *	0.19	0.05	1.06 *^#^	0.21	0.06	*p* = 0.036
IL-6 [pg/mL] after anaerobic test	baseline	1.47	0.45	0.12	1.57	0.63	0.17	*p* = 0.5577
	16-weeks	0.97 ^#^	0.27	0.08	1.05 ^#^	0.31	0.09	*p* = 0.9154

x—mean; SD—standard deviation; SE—standard error; * Statistically significant intergroup differences (*p* < 0.05); ^#^ Statistically significant differences compared with the initial value (*p* < 0.05).

**Table 11:** The concentration of Interleukin 8 (IL-8) pre- and post-aerobic test and pre- and post- anaerobic test at follow-up measurements during supplementation intervention.

Indicator/Measurement		Probiotic Group (P)			Control Group (C)			*p*
		x	SD	SE	x	SD	SE	
IL-8 [pg/mL] before aerobic test	baseline	6.75 *	1.12	0.31	4.70 *	1.40	0.39	*p* = 0.0086
	16-weeks	7.68	1.80	0.50	7.10 ^#^	1.10	0.30	*p* = 0.1814
IL-8 [pg/mL] after aerobic test	baseline	7.48	3.20	0.89	5.41	1.23	0.34	*p* = 0.2334
	16-weeks	8.48	1.36	0.38	8.73 ^#^	2.50	0.69	*p* = 0.1814
IL-8 [pg/mL] before anaerobic test	baseline	7.38	2.65	0.73	6.70	1.00	0.28	*p* = 0.9442
	16-weeks	6.87	1.39	0.39	6.57	1.03	0.28	*p* = 0.7893
IL-8 [pg/mL] after anaerobic test	baseline	6.71	1.39	0.38	6.11	0.41	0.11	*p* = 0.9442
	16-weeks	6.65	2.02	0.56	5.48	2.38	0.66	*p* = 0.7893

x—mean; SD—standard deviation; SE—standard error; * Statistically significant intergroup differences (*p* < 0.05) ^#^ Statistically significant differences compared with the initial value (*p* < 0.05).

**Table 12:** The concentration of Interleukin 10 (IL-10) pre- and post-aerobic test and pre- and post- anaerobic test at follow-up measurements during supplementation intervention.

Indicator/Measurement		Probiotic Group (P)			Control Group (C)			*p*
		x	SD	SE	x	SD	SE	
IL-10 [pg/mL] (before aerobic test)	baseline	0.48	0.37	0.10	0.54	0.25	0.07	*p* = 0.85
	16-weeks	0.45	0.28	0.08	0.36	0.19	0.05	*p* = 0.115
IL-10 [pg/mL] (after aerobic test)	baseline	0.76	0.72	0.20	0.57	0.35	0.10	*p* = 0.266
	16-weeks	0.47 *^#^	0.56	0.15	0.29 *	0.11	0.03	*p* = 0.023
IL-10 [pg/mL] (before anaerobic test)	baseline	0.70 *	0.43	0.12	0.39 *	0.18	0.05	*p* = 0.047
	16-weeks	0.44	0.25	0.07	0.35	0.19	0.05	*p* = 0.12
IL-10 [pg/mL] (after anaerobic test)	baseline	0.54 *	0.33	0.09	0.31 *	0.18	0.05	*p* = 0.025
	16-weeks	0.48 *	0.34	0.09	0.29 *	0.14	0.04	*p* = 0.023

x—mean; SD—standard deviation; SE—standard error; * Statistically significant intergroup differences (*p* < 0.05); ^#^ Statistically significant differences compared with the initial value (*p* < 0.05).

**Table 13:** Total oxidative status (TOS) of the blood plasma pre- and post-aerobic test and pre- and post- anaerobic test at follow-up measurements during supplementation intervention.

Indicator/Measurement	Probiotic Group (P)				Control Group (C)			*p*
	x		SD	SE	x	SD	SE	
TOS—before aerobic test	baseline	352.9	226.6	62.85	562.9	406.4	112.7	*p* = 0.11
	4-weeks	536.6	266.9	74.02	624.13	1786	495.4	*p* = 0.19
	12-weeks	492.4	192.3	53.34	480.9	40.30	11.18	*p* = 0.833
	16-weeks	498.6	242.4	67.23	625.1	365.6	101.4	*p* = 0.309
TOS—after aerobic test	baseline	546.9	284.7	78.96	757.1	197.7	54.83	*p* = 0.22
	4-weeks	616.0	318.5	88.34	620.13	340.0	94.29	*p* = 0.45
	12-weeks	1023	722.7	200.4	957.3	370.2	102.7	*p* = 0.772
	16-weeks	692.1	470.9	130.6	770.3	153.8	42.65	*p* = 0.574
TOS—before anaerobic test	baseline	663.7 *	433.3	120.2	783.9 *	272.3	75.52	*p* = 0.04
	4-weeks	497.7 *	266.7	73.97	711.2 *	280.0	77.67	*p* = 0.048
	12-weeks	536.8 *	247.7	68.69	652.5 *	189.9	52.66	*p* = 0.019
	16-weeks	484.6 *^#^	235.8	65.41	918.1 *	295.6	81.98	*p* = 0.024
TOS—after anaerobic test	baseline	643.1 *	391.5	108.6	740.9 *	245.3	68.04	*p* = 0.045
	4-weeks	402.8 *	191.2	53.03	1635 *	1426	395.7	*p* = 0.005
	12-weeks	625.1	568.7	157.7	662.6	271.6	75.32	*p* = 0.83
	16-weeks	435.9 *^#^	256.2	71.06	751.4 *	292.6	81.14	*p* = 0.034

x—mean; SD—standard deviation; SE—standard error; * Statistically significant intergroup differences (*p* < 0.05); ^#^ Statistically significant differences compared with the initial value (*p* < 0.05).

**Table 14:** Total anti-oxidant (TAS) status of the blood plasma pre- and post-aerobic test and pre- and post- anaerobic test at follow-up measurements during supplementation intervention.

Indicator/Measurement		Probiotic Group (P)			Control Group (C)			*p*
		x	SD	SE	x	SD	SE	
TAS—before aerobic test	baseline	302.3	36.39	10.09	319.6	34.95	9.69	*p* = 0.229
	4-weeks	305.7	30.29	8.40	321.2	28.91	8.02	*p* = 0.196
	12-weeks	302.0	50.68	14.06	313.7	27.77	7.70	*p* = 0.470
	16-weeks	303.7	29.58	8.20	289.1	31.91	8.85	*p* = 0.237
TAS—after aerobic test	baseline	331.3	39.04	10.83	326.5	33.13	9.19	*p* = 0.738
	4-weeks	307.1	38.83	10.77	294.8	36.01	9.99	*p* = 0.41
	12-weeks	338.4	51.21	14.20	290.9	27.28	7.56	*p* = 0.686
	16-weeks	299.0	47.75	13.24	304.5	9.71	2.69	*p* = 0.68
TAS—before anaerobic test	baseline	302.8	34.02	9.44	343.4	22.03	6.11	*p* = 0.23
	4-weeks	308.9	40.29	11.17	309.4	37.06	10.28	*p* = 0.970
	12-weeks	291.0	45.77	12.69	298.2	51.16	14.19	*p* = 0.708
	16-weeks	279.8	44.12	12.24	292.7	52.30	14.51	*p* = 0.11
TAS—after anaerobic test	baseline	328.7	59.28	16.44	329.7	57.11	15.84	*p* = 0.42
	4-weeks	314.5	40.79	11.31	363.3	22.66	6.28	*p* = 0.06
	12-weeks	308.3	56.69	15.72	304.7	45.20	12.54	*p* = 0.860
	16-weeks	301.2	14.71	4.08	299.9	44.11	12.23	*p* = 0.925

x—mean; SD—standard deviation; SE—standard error.

## Data Availability

Not applicable.
